# Supplementary material for: U-CAN-seq: A Universal Competition Assay by Nanopore Sequencing
Source: Viruses. 2024 Apr 19;16(4):636. doi: 10.3390/v16040636 (PMC11054411; doi:10.3390/v16040636)
Supplement: Supplementary file 1 [file viruses-16-00636-s001.zip › viruses-2919005-suppl.pdf]

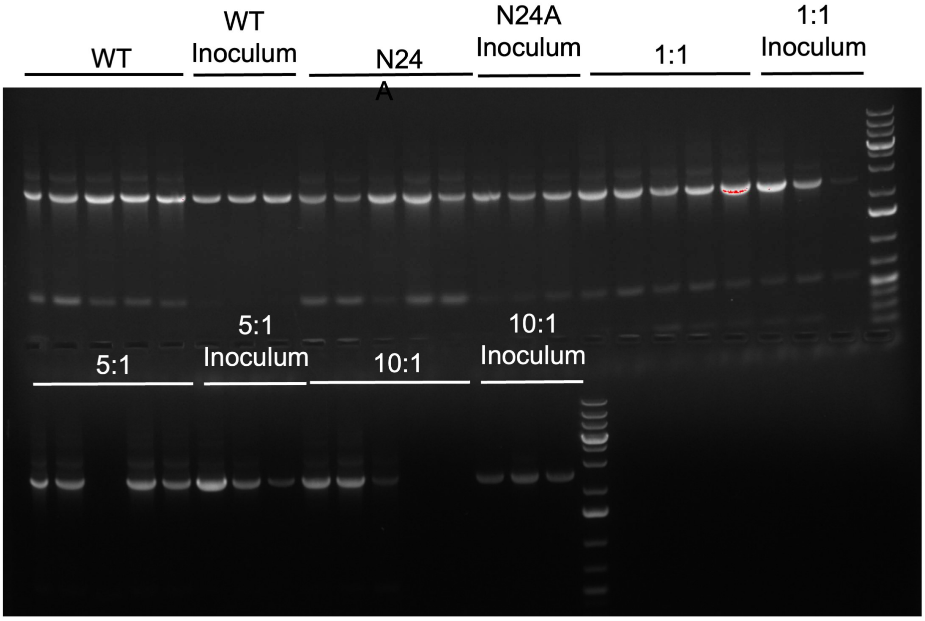

Figure S1. Consistent PCR amplification from inocula and supernatants of infected cells.

```
for file in
/Users/jenloome/Desktop/Desktop_Jennifer’s_MacBook_Air/Notebooks_and_backups/0148_AV_Competition_assay_gro
wth_curve_structure_scr_3/Loome_p9g_raw_reads/*.fastq; do { echo "$file"; echo "WT,"$(grep -o
"ACGTGGACATAGACGCTGACAGCGCCTTTTT" "$file" | wc -l); echo "scrSL3,"$(grep -o
"ATGTAGACATAGACGCTGACAGCGCCTTTCT" "$file" | wc -l); } >> 0148_AV_scrSL3_output.csv; done
```

-Directory where your fastq files are at (on mac is probably /User/UserName/Downloads/PlasmidsourausRawReadDirectory/*.fastq), can be found in terminal by typing “pwd” while in that directory

-Name of string one (appears in the .csv file)

-First string that you are searching for

-Name of string two (appears in the .csv file)

-Second string that you are searching for

-Whatever you’d like to call your output file

Figure S2. Unix code for k-mer search.
